# Eye Recognition by YOLO for Inner Canthus Temperature Detection in the Elderly Using a Transfer Learning Approach

**DOI:** 10.3390/s23041851

**Published:** 2023-02-07

**Authors:** Malak Ghourabi, Farah Mourad-Chehade, Aly Chkeir

**Affiliations:** Computer Science and Digital Society (LIST3N), University of Technology of Troyes, 10000 Troyes, France

**Keywords:** deep learning, elderlies, infectious diseases, physical frailty, temperature detection, thermal image processing, transfer learning, YOLO

## Abstract

Early detection of physical frailty and infectious diseases in seniors is important to avoid any fatal drawback and promptly provide them with the necessary healthcare. One of the major symptoms of viral infections is elevated body temperature. In this work, preparation and implementation of multi-age thermal faces dataset is done to train different “You Only Look Once” (YOLO) object detection models (YOLOv5,6 and 7) for eye detection. Eye detection allows scanning for the most accurate temperature in the face, which is the inner canthus temperature. An approach using an elderly thermal dataset is performed in order to produce an eye detection model specifically for elderly people. An application of transfer learning is applied from a multi-age YOLOv7 model to an elderly YOLOv7 model. The comparison of speed, accuracy, and size between the trained models shows that the YOLOv7 model performed the best (Mean average precision at Intersection over Union of 0.5 (mAP@.5) = 0.996 and Frames per Seconds (FPS) = 150). The bounding box of eyes is scanned for the highest temperature, resulting in a normalized error distance of 0.03. This work presents a fast and reliable temperature detection model generated using non-contact infrared camera and a deep learning approach.

## 1. Introduction

According to the European Union data, by 2050, the number of elderly people will increase by 70% for those over 65 years old and 170% for those over 80 [1]. In addition, the elderly population is severely affected by the COVID-19 crisis, due to the higher mortality risk upon exposure to a viral infection. It was noticed that in France, upon facing the COVID-19 pandemic, the most affected people where those aged 65 years old and over, with at least 92% of COVID-19 cases [2]. Moreover, viral infections have stronger and more fatal effects on seniors [3]. For example, sometimes COVID-19 surpasses the common cold, with its serious symptoms characterized by lung infection, a major cause of respiratory distress syndrome (ARDS) [4].

Identifying physical frailty in the elderly has become a vital issue to keep them as far away as possible from being exposed to the virus, as their chance of survival after exposure is very low. However, early detection allows corrective actions as soon as possible and helps reduce contamination [5].

One of the indicators that help in detecting viral infections is elevated body temperature [6]. A thermal camera, being a fast and reliable non-contact temperature measurement device, is considered a better option than contact medical thermometers. The U.S. Food and Drug Administration (FDA), in their article entitled “The Medical Devices’ Safety of Non-contact Temperature Assessment Devices During the COVID-19 Pandemic”, stated that infrared thermographic systems help in preventing virus transmission, because they are non-contact temperature screening devices [7]. Additionally, a review on the medical applications of infrared thermography mentioned that the temperature of the inner canthi of the eyes is the most accurate temperature to be measured using a thermal camera. This is due to its correlation with the temperature of the axilla measured by a conventional thermometer [8]. We know that the internal carotid, the artery that supplies the interior part of the brain, passes underneath the inner canthus region, making it the most reliable region for temperature detection by thermal infrared camera [9]. Moreover, a study mentioned in [8] has shown that a temperature above 37.5 °C, with ±0.5 °C tolerance, is considered a possible indication of fever.

In order to detect the inner canthus of the eyes, one should start first by locating the eyes in an image. This can be done by implementing object detection techniques which are widely used in computer vision tasks. These techniques are either of a traditional machine-learning nature or a deep-learning nature. Object detection deep-learning methods have better performance in the case of large-scale data training. From these methods, we have R-CNN, Fast R-CNN, Faster R-CNN, Mask R-CNN and YOLO [10]. YOLO is a real-time object monitoring algorithm, due to its small size and fast operational speed in comparison to other object detection alternatives. In addition, YOLO can detect objects in videos, making it a better choice in real-time detection scenarios [11].

Several techniques that aim for eye detection in thermal images were proposed in the literature. For example, Hussein et al. worked on a training cascade-based classifier, feeding it by Haar, histogram of oriented gradient (HoG), and local binary patterns (LBP) features from 1000 images taken from the Natural Visible and Infrared Facial Expression Database (NVIE). Among the three types of features, HoG features fed to the cascade classifier achieved the highest precision and recall rates, at 98.8% and 92.6%, respectively [12]. Another group of researchers attempted to detect the eye frame and inner canthus in 15 images of different face orientations. This was carried out by applying face segmentation and rotation of the face into a straight view, followed by using facial proportion to locate the eyes, resulting in an accuracy of 80%. After locating the eyes, they searched the frame for the highest intensity to localize the inner canthus, ending with 100% localization accuracy. However, this algorithm lacks reliability in cases of longer face height and in cases of neck presence in the image, for example, which resulted in an error in proportions. Accordingly, the eye frame will not be correctly localized, causing incorrect inner canthus detection [13]. Additionally, Knapik et al., in their paper entitled “Fast Eyes Detection in Thermal Images”, presented a pre-processing image technique that mainly converts the low-dynamic range thermal image into a high-dynamic range image for detail enhancement followed by the use of scale-invariant feature transform (SIFT). Their final detections were carried out using the bag of visual words clustering approach. The work achieved precision and recall of 96% and 97%, respectively, when testing the YOLOv3 deep learning model trained on 62 samples [14].

Besides the mentioned research, some work has been dedicated to inner canthus temperature detection, such as the work presented in [15], which trained two versions of the “you only look once” (YOLO) object detection algorithm to detect eyes’ inner canthi region. YOLOv4 and YOLO-Tiny versions of YOLO were trained using 606 thermal images of 35 individuals, resulting in a precision score of 0.94 and 0.99, respectively, and the same recall score of 0.99. Furthermore, Budzan et al. worked on face and eye localization for inner canthus temperature measurement by implementing randomized Hough transform for ellipses detections (the two eyes in this case). The method was tested on 125 thermal images of faces and resulted in an average accuracy of 97.3% [16]. Ferrari et al. proposed an algorithm based on the OpenPose detector to detect inner canthi locations, followed by application of a 3D Morphable Face Model to refine the detections. The model runs at a speed of nine frames per second (FPS), which is considered low compared to other approaches. Their work was performed by training the OpenPose detector on visible and not thermal images, making it not reliable enough [17]. Finally, the most recent attempt was that of Lazri et al. [18], who worked on detecting inner canthi and nostrils to measure body temperature and respiration rate. They used the pre-trained single shot multibox detector (SSD) to detect faces in thermal images, then they searched for the landmarks using Kazemi and Sullivan’s publicly available algorithm. The model was tested on 36 thermal images, plus four processed image types per one thermal image, and fairly detected the inner canthi and nostrils with a speed of 146 FPS. The detection was accomplished only in frontal faced images along with the confusion of eyebrows region. These limitations are because both SSD and landmarks detection models were initially trained on visible frontal images only.

This paper presents an original approach for detecting the eye region of the face using YOLO’s object detection method. The different architectures of YOLO versions 5,6 and 7 are trained and compared in this work. At first, multi-age images collected from online image datasets and captured thermal images are used for the training and testing processes. Then, transfer learning is carried out, starting from the multi-age model weights, to train a new model on the limited number of elderly faces in thermal images. This is followed by an inner canthus temperature extraction method.

The rest of the paper is organized as follows. Section 2 introduces the materials and describes the methodology. Section 3 shows the results, followed by a discussion in Section 4. A conclusion with work perspectives is finally presented in Section 5.

## 2. Materials and Methods

**Figure 1:**
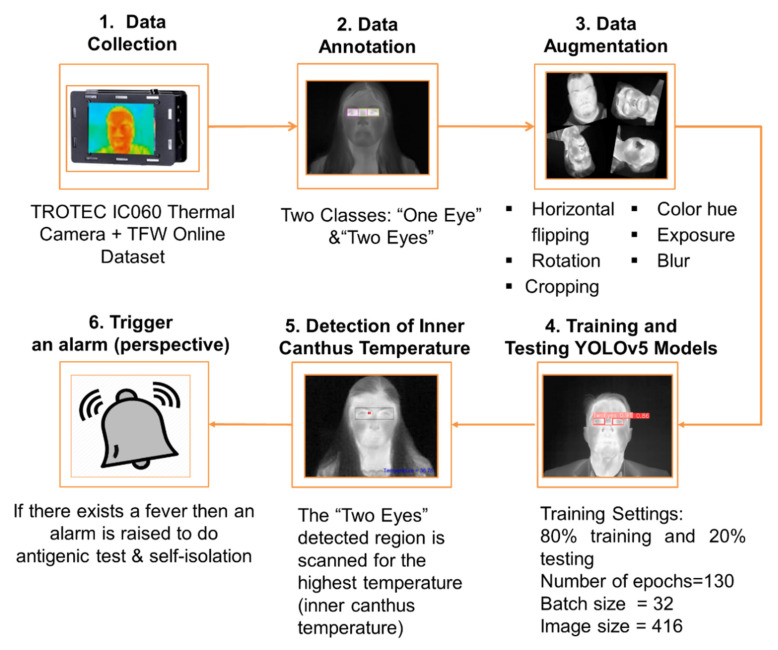
The methodology followed in this work.

The general methodology of this work consists of several steps. The first step is the data collection and preparation procedure, followed by labelling the eyes in the dataset. After data annotation, images are introduced to data augmentation techniques to increase their number and avoid the model’s overfitting. Then, training and testing of YOLO different models is carried out. Finally, the detected eye area is scanned for the inner canthus temperature. These steps are applied once on a multi-age dataset and another time on an elderly dataset. Figure 1 shows a flowchart of the methodology proposed in our work wherein the final step will be performed later, retrospectively.

### 2.1. Datasets Collection

#### 2.1.1. The Multi-Ages Dataset

The multi-age dataset consists of 1827 thermal images, a combination of thermal images captured in our lab (104 images of 11 individuals) and the TFW testing indoor dataset (1723 images of 23 individuals). The merging of two different datasets is performed in order to obtain larger and more varied samples of face thermal images. This method will ensure a more generalized model [19].

**Figure 2:**
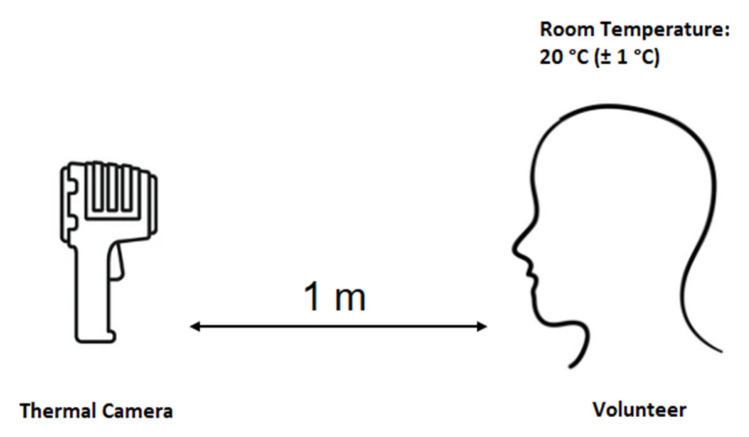
Experimental setup of thermal image capturing.

Data collection in the lab is carried out using a TROTEC IC060 thermal camera with a resolution of 152 × 115. The equipment’s sensor is an uncooled microbolometer focal plane array with a minimum focus distance = 0.1m and a spatial resolution = 2.2 mrad. The captured images in SAT file format are transferred to the PC using a USB data cable. TROTEC provides ICReport software which allows exportation of the images and their temperature data in JPG and xlsx Excel spreadsheet formats, respectively. A total of 104 images of 11 individuals are captured. Five males and six females aged between 22 and 30 contributed to data collection. The images are captured in our lab, with the camera being parallel to the participants and placed at a distance of 1m [20]. The ambient temperature is maintained at 20 °C (±1 °C) and measured using a thermometer. All our participants were asked to spend 15 min in the room in order to prevent the thermal effect of the external environment, and the same setup conditions were preserved for all captures. The experiment was explained to the volunteers, who agreed to let their images be utilized for research and publication purposes. Figure 2, below, shows the experimental setup.

The second dataset, named the “annotated thermal faces in the wild dataset (TFW)”, is a visual and thermal dataset containing images of 147 subjects of different age groups. It counts 9982 images collected in controlled and uncontrolled and indoor and outdoor environments. The indoor environment’s temperature is maintained at 25 °C. The images are taken using FLIR T540 thermal camera with a resolution of 464 × 348 pixels. Note that the TFW dataset is originally split to training, testing, and validation in order to train a YOLOv5 model to detect individuals’ faces and their landmarks (pupils, nose, and borders of mouth). Therefore, we have chosen its indoor unlabeled testing dataset, which contain 2160 images of 30 individuals. However, 7 individuals are wearing eyeglasses; accordingly their images are eliminated [21]. This leaves the dataset with 1723 images of 23 individuals with different head rotations (upward, downward, left and right).

#### 2.1.2. The Elderly Dataset (TFW, Tufts, IRDatabase)

The preparation of the elderly dataset is done by collecting images from three different online thermal datasets: TFW [21], Tufts [22,23] and IRDatabase [24]. TFW database is collected in the Institute of Smart Systems and Artificial Intelligence, Nazarbayev University, Kazakhstan. The second database, IRdatabase is collected in RWTH Aachen University in Germany. The third database, Tufts, is built in Tufts University, Boston, USA. The elderly participants in the three previous datasets are staff, faculty members, and/or their family members, alongside students’ family members. This results in a total of 656 images for 20 individuals aged 60 and above. The distribution of the sources of the database is presented in Table 1.

### 2.2. Dataset Preparation

#### 2.2.1. Data Annotation

**Figure 3:**
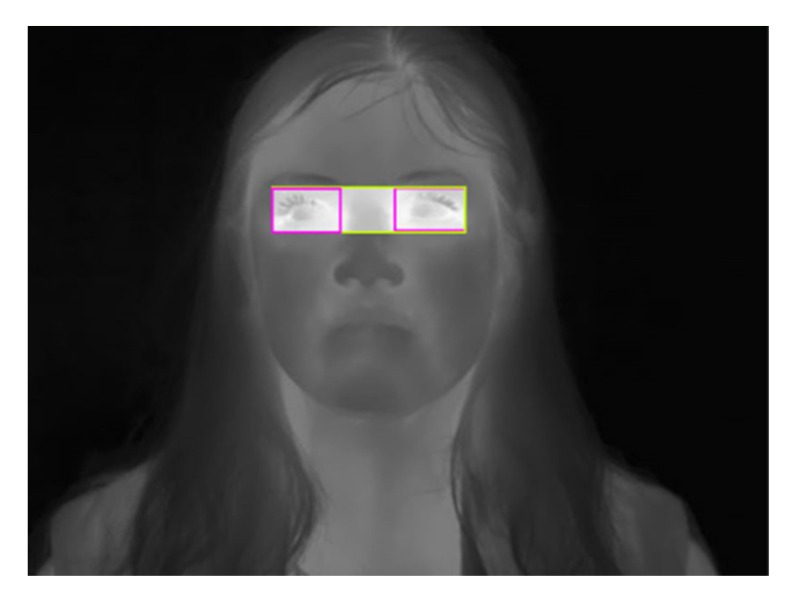
Example of the labeled image; pink box: one eye class, yellow box: two eyes class.

We used Roboflow online tool to label our data [25]. The labelling consists of two classes: one eye and two eyes. The class one eye includes each eye in two separate bounding boxes; however, the class two eyes includes both eyes in the same bounding box. Figure 3 shows an example of a labeled image where the pink boxes refer to the one eye class and the yellow box refers to the two eyes class.

For the elderly dataset, the labeling is limited to the two eyes class, which is simply called eyes. This is due to using the two eyes box exclusively later in the methodology.

#### 2.2.2. Data Augmentation

After labeling the 1827 multi-age dataset images and the 656 elderly dataset ones, we integrate different data augmentation techniques using the Roboflow data augmentation tool in order to increase the size of our dataset [25,26].

The techniques applied are:

- Horizontal flipping;
- Rotations;
- Cropping;
- Saturation;
- Exposure; and
- Blur.

**Figure 4:**
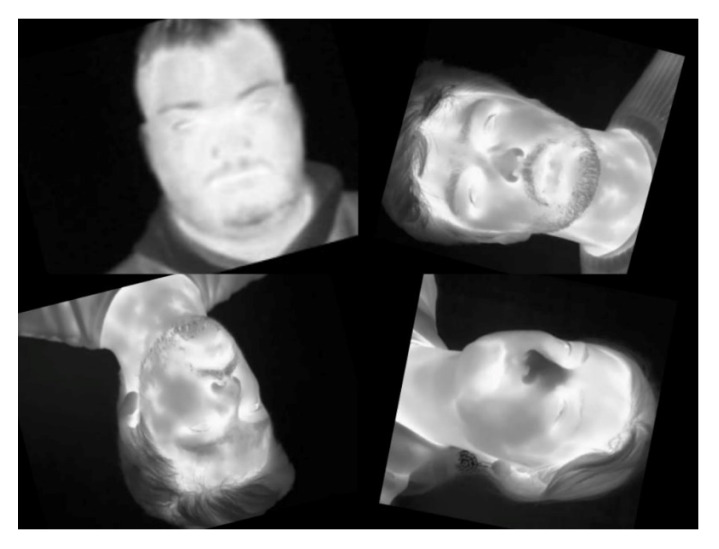
Examples of the augmented images.

Consequently, we had a total of 4255 images for the multi-age dataset and 1,791 images for the elderly dataset. Some of the augmented photos are presented in Figure 4. The rotations displayed in Figure 4 are done in order to allow the model to learn the different possible face positions of the subjects. This way, it can detect the subject’s eyes whether he or she is lying down or whether the thermal camera is attached to the ceiling.

### 2.3. YOLO: Algorithm and Versions

“You only look once” (YOLO) is a unified single convolutional neural network (CNN) model proposed by Redmon et al. for object detection in images [27]. In this work, YOLO’s open-source CNN based software is chosen due to its high detection accuracy and satisfactory computational complexity.

**Figure 5:**
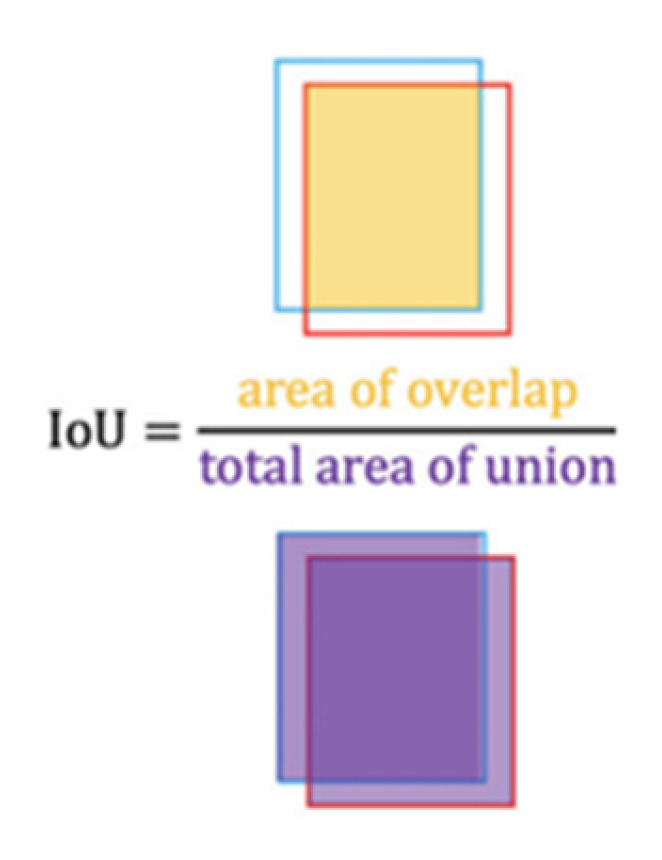
Intersection over union (IoU) is the division of the overlapping area by the total union area between the ground truth bounding box and the predicted one [28].

The YOLO model is trained on the COCO dataset to detect multiple bounding boxes with their class probabilities [27]. It divides the image into an S × S grid, wherein each grid cell predicts B bounding boxes along with their confidence scores. Equation (1) shows how the confidence score for each bounding box is calculated.

(1) $${\mathrm{Confidence}\;\mathrm{Score}}_{\mathrm{Object}}=\mathrm{Pr}\left(\mathrm{Object}\right){*\;\mathrm{IoU}}_{\mathrm{prediction}}^{\mathrm{truth}},$$

where $\mathrm{Pr}\left(\mathrm{Object}\right)$ shows the probability that the cell contains an object and ${\mathrm{IoU}}_{\mathrm{prediction}}^{\mathrm{truth}}$ is the intersection of the union between the detected box and the ground truth (Figure 5). If the cell does not contain an object, then the Pr(Object) should be a zero leading to zero confidence score. On the contrary, the aim is to have the ${\mathrm{IoU}}_{\mathrm{prediction}}^{\mathrm{truth}}$ equal to the ${\mathrm{Confidence}\;\mathrm{Score}}_{\mathrm{Object}}$.

Besides the confidence score above, each grid cell containing an object predicts C conditional class probabilities, $\mathrm{Pr}\left({\mathrm{Class}}_{i}|\mathrm{Object}\right)$. This metric shows the probability that the detected object belongs to class i.

During testing of the model, a class-specific confidence score for each bounding box is calculated, as shown in Equation (2).

(2) $$\mathrm{Pr}\left({\mathrm{Class}}_{i}|\mathrm{Object}\right)*\mathrm{Pr}\left(\mathrm{Object}\right){*\;\mathrm{IoU}}_{\mathrm{prediction}}^{\mathrm{truth}}=\mathrm{Pr}\left({\mathrm{Class}}_{i}\right){*\;\mathrm{IoU}}_{\mathrm{prediction}}^{\mathrm{truth}},$$

**Figure 6:**
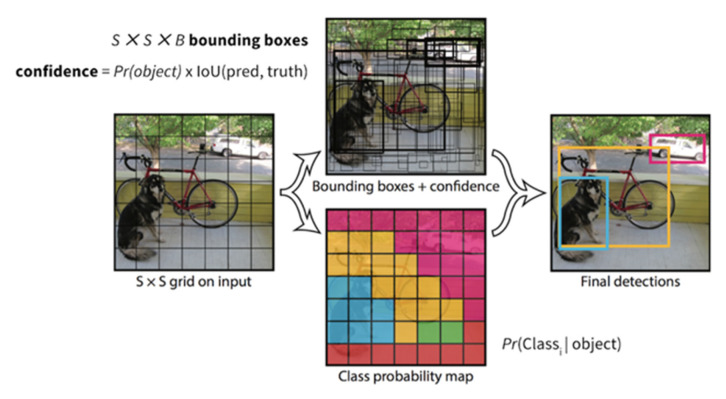
YOLO workflow [23].

Figure 6 shows the workflow of the YOLO model, in which each image contains S × S × B bounding boxes. Each box has the following predictions: the center coordinates (x,y), weight, height, 1, confidence score, and C, conditional class probabilities [27].

**Figure 7:**
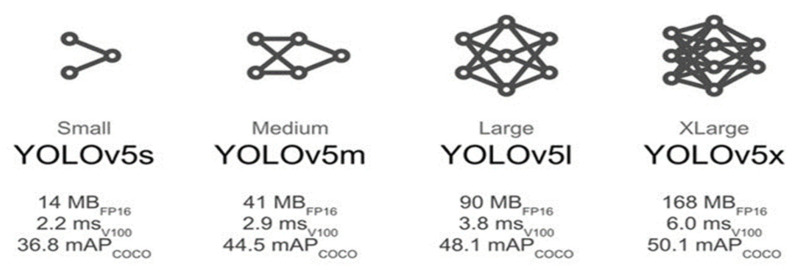
YOLOv5 different model sizes, where FP16 is the half floating-point precision, v100 stands for the inference time in milliseconds on the NVIDIA V200 GPU, and mAP (mean average precision) is calculated according to the original COCO dataset [30].

There are seven main YOLO versions: the first YOLO version, YOLOv2, YOLOv3, YOLOv4, YOLOv5, YOLOv6 and YOLOv7. Both YOLOv5 and YOLOv6 have models of different sizes, whereas YOLOv5 comes with five different model sizes from Nano to X-large (YOLOv5n, YOLOv5s, YOLOv5m, YOLOv5l, and YOLOv5xl), as shown in Figure 7. Moreover, YOLOv6 is of two sizes, nano or small (to date); larger sizes are still in development. The choice of the model size is a tradeoff between accuracy and computational power. Figure 7 shows how larger models have greater mean average precision (mAP) scores when trained and tested on the COCO dataset [29].

**Figure 8:**
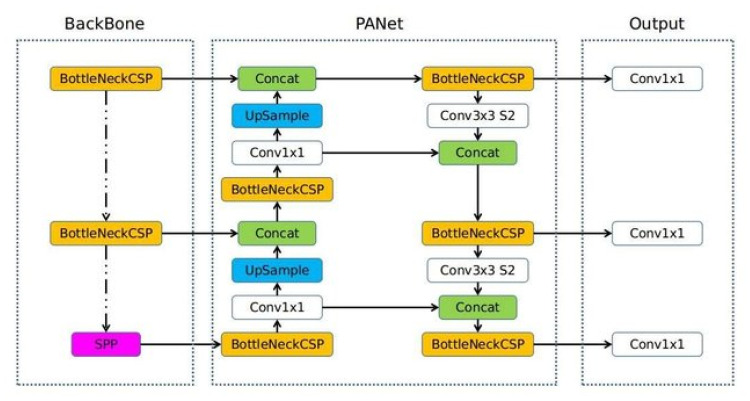
YOLOv5 model architecture [30].

In this study, we train the three most recent YOLO versions, 5, 6, and 7. The YOLOv5 model architecture shown in Figure 8 comprises three independent parts: the backbone, neck and head. The backbone network is responsible for feature extraction. Then, the features map is introduced to the neck which in turn detects the bounding boxes. Finally, the head gives the detection results (class, confidence score, location(s) and size(s) of the bounding box(es)) [31]. Another advantage is its ability to enhance the training data, where the data loader of YOLOv5, for example, applies three types of data enhancement: color space adjustment, scaling and mosaic enhancement [30]. YOLOv6 offers a hardware-friendly design and high performance dedicated to industrial applications. Changes are made in the backbone and neck of YOLOv5, in order to meet these criteria. There exist, as of now, the YOLOv6-nano and YOLOv6-small model sizes, while other sizes are still in development [32]. YOLOv7, unlike YOLOv5, does not use the ImageNet pre-trained backbones. However, the models are trained on the COCO dataset entirely. There are two major changes in the YOLOv7 architecture: The first is the presence of a computational block in its backbone named E-ELAN (extended efficient layer aggregation network), which allows the framework to learn better. Secondly, the YOLOv7 introduces new BoF (bag of freebies) methods that enhance performance without increasing training costs [33].

### 2.4. Training Settings

**Figure 9:**
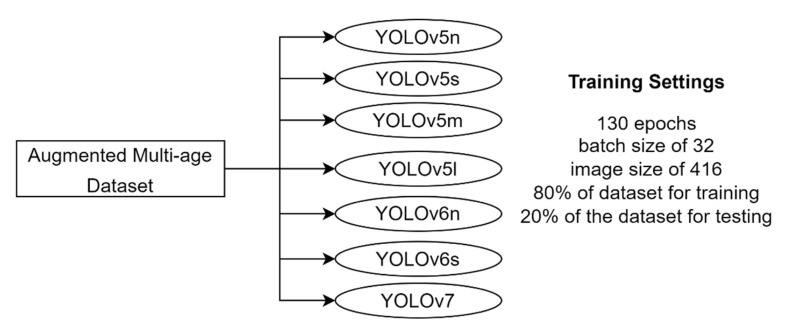
Training settings for the augmented multi-age dataset: different YOLO models trained with 130 epochs, a batch size of 32, an image size of 416, and an 80–20% train-test split.

The training settings of the augmented multi-age dataset are detailed in Figure 9, where seven different models were established.

**Figure 10:**
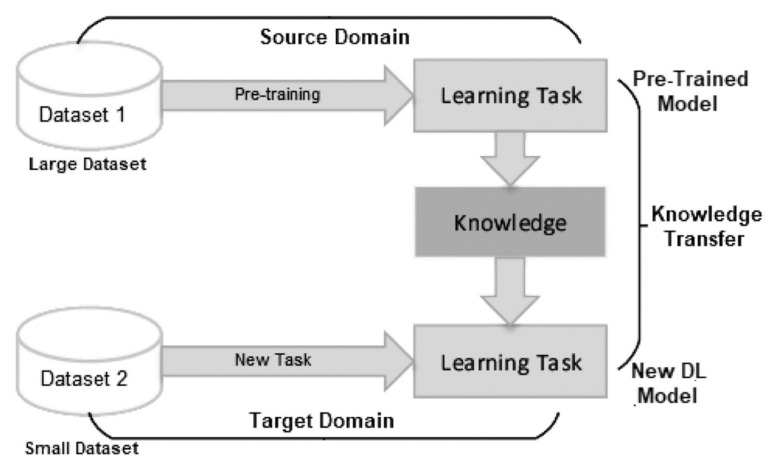
Transfer learning structure. DL: deep learning [34].

**Figure 11:**
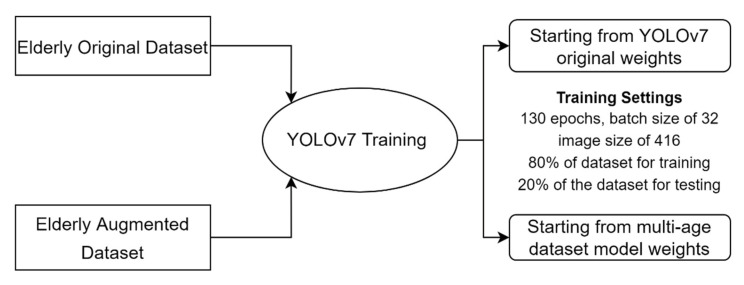
Training of elderly original and augmented datasets using YOLOv7 original weights and YOLOv7 multi-age dataset model weights.

For the elderly dataset, we have used both the original and augmented versions separately to train different models of YOLOv7 and compare among them. The training consisted of two stages: the first started from the original YOLOv7 weights; however, the second started from the weights of YOLOv7 model trained on the augmented multi-age dataset (i.e., transfer learning). A general structure of transfer learning is represented in Figure 10, wherein the knowledge is transferred from a model trained on the large dataset to a new model [34]. Figure 11 shows the training settings for the elderly dataset.

**Figure 12:**
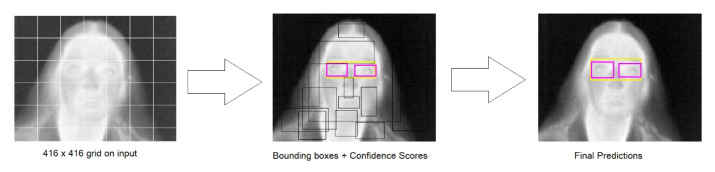
The models’ processing steps on the input image.

Figure 12 shows how the trained models process the input image. First, the input image splits into 416 × 416 grids. Second, bounding boxes are predicted by each grid, along with their confidence scores and class probabilities. Finally, classes are detected on the output by tracing bounding boxes on the originally input image.

### 2.5. Blindfold Testing

The blindfold testing approach is adopted to examine the eye detection model further. This approach is basically training the model on a number of faces and testing it on new faces. In this way, the model is tested for its ability to work in a practical scenario where it will be subjected to varied images.

In order to do so, the TFW elderly dataset is used for training (504 images), and the IRDatabase and TUFTS elderly datasets are used for testing (152 images). The previous data augmentation techniques are applied to the training dataset, where its size increased by three times. Additionally, the same training settings used before (130 epochs, a batch size of 32 and an image size of 416) are applied for the blindfold model. The training started from the weights of YOLOv7 model trained on the augmented multi-age dataset.

### 2.6. Detection of Inner Canthus Temperature

After detecting the two eyes in a bounding box, this region is scanned for the highest temperature. The highest temperature corresponds to the inner canthus of the eye. As mentioned in the introduction, the inner canthus is the optimal region to detect the body’s temperature using an infrared thermometer [9]. The process of detecting the temperature is done using a code generated in MATLAB R2021a, wherein the temperature data is scanned for the highest temperature, starting from the location of the upper left edge of the bounding box to its lower right edge. The eye region, the inner canthus point, and the temperature are then presented on the image processed.

In order to further validate the detected inner canthus point, we followed the following procedure:

1. Run the algorithm of inner canthus and temperature detection on a number of the images collected in our lab.
2. Register the automatically detected pixel (q) coordinates ($q_{x},q_{y}$) for each image.
3. Locate manually the inner canthi for the same images and extract their pixel (p) coordinates ($p_{x},p_{y}$).
4. Normalize the q and p pixel coordinates to the image size 152 × 116 (Equations (3)–(6)) in order to obtain a normalized metric ($q_{x_{\mathrm{norm}}},p_{x_{\mathrm{norm}}},q_{y_{\mathrm{norm}}}{,p}_{y_{\mathrm{norm}}})$.
  (3) $$q_{x_{\mathrm{norm}}}=\frac{q_{x}}{152}$$
  (4) $$p_{x_{\mathrm{norm}}}=\frac{p_{x}}{152}$$
  (5) $$q_{y_{\mathrm{norm}}}=\frac{q_{y}}{116}$$
  (6) $$p_{y_{\mathrm{norm}}}=\frac{p_{y}}{116}$$
5. Calculate the Euclidean distance (d) between the manually and automatically detected inner canthus of each image using Equation (7) [35].
6. Calculate the mean of the distances (d).
  (7) $$d=\sqrt{\Delta x^{2}+\Delta y^{2}}$$
  where
  $$\Delta {x=q}_{\mathrm{xnorm}}{-\;p}_{\mathrm{xnorm}}\;\mathrm{and}\;\Delta {y=q}_{\mathrm{ynorm}}{-\;p}_{\mathrm{ynorm}}$$

## 3. Results

### 3.1. Trained Models Results

The training and analysis of results of YOLO models are performed on Google Colab Python-based virtual machine using a TensorFlow library for machine learning and artificial intelligence [36]. The results of testing the trained models are displayed in Table 2. The metrics chosen to evaluate the different trained models are precision (P), recall (R), and mean average precision (mAP). Precision shows the ability of the model to detect only correct predictions (true positives). A prediction is said to be a true positive (TP) if the IoU of the bounding box is greater or equal to 0.5. Otherwise, if the IoU is less than 0.5 or a duplicated bounding box exists for the same object, then the prediction is denoted as a false positive (FP). On the other hand, a false negative (FN) occurs when there exists a ground truth bounding box but the model did not predict it. Below, Equations 8 and 9 are those of P and R, respectively.

(8) $$P=\frac{\mathrm{TP}}{\mathrm{TP}+\mathrm{FP}}$$

(9) $$R=\frac{\mathrm{TP}}{\mathrm{TP}+\mathrm{FN}}$$

The F1-score is calculated from the precision and recall. The F1-score is a machine learning evaluation metric that measures the model’s accuracy by taking into account both the false positives and false negatives of the tested model, and is calculated as shown in Equation (10) [37].

(10) $$F1\text{-}\mathrm{Score}=2\;\times \frac{P\;\times \;R}{P+R}$$

Equation (11) below expresses mAP, which is defined as the area under the precision–recall curve. This metric is standardly used in object detection applications and helps in analyzing the model’s accuracy. In our case, mAP is evaluated under the condition of IoU ≥ 0.5, hence it is denoted as mAP@.5 [28].

(11) $$\mathrm{mAP}=\frac{\sum_{q=1}^{Q}\mathrm{AveP}(q)}{Q}$$

where Q signifies the number of queries in the set and AveP(q) signifies the average precision for a given query (q).

**Figure 13:**
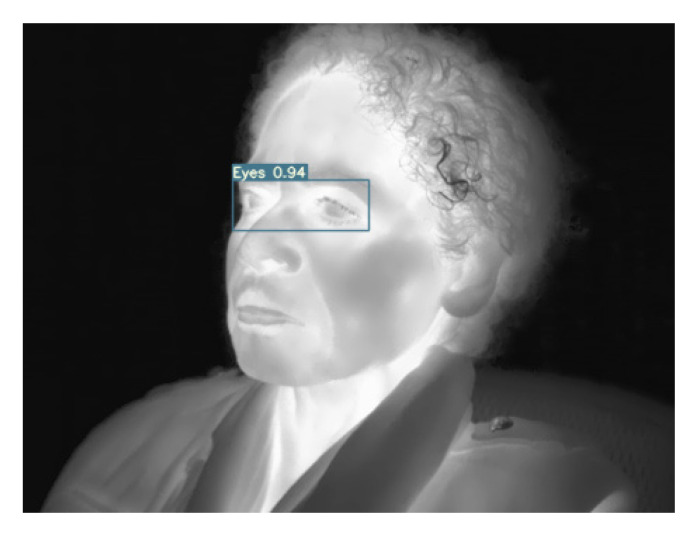
Detection of eyes in a senior face image using YOLOv7 elderly transferred model.

An example of eye detection using YOLOv7 elderly model, which is a result of transferring the augmented multi-age dataset model, is presented in Figure 13.

In order to test the inference of the trained models, we have recorded a video of 13 s of a volunteer looking at the camera and turning slightly to the left. Table 3 shows the size, inference time and frame processed per second (FPS) of the different training models. FPS is the number of image frames per second processed by the model. The more processed frames per second, the faster the model infers. The FPS is found by calculating the reciprocal of the inference time (Equation (12)) [38].

(12) $$\mathrm{FPS}\;(\mathrm{frames}\;\mathrm{per}\;\mathrm{second})=\frac{1}{\mathrm{inference}\;\mathrm{time}(s)}$$

YOLOv6, as shown in Table 1, had the lowest mAP@.5 score; therefore, it is eliminated and not tested for inference.

The model size metric in megabytes (MB) is considered in order to take it into consideration later on when developing an embedded system of the model.

**Figure 14:**
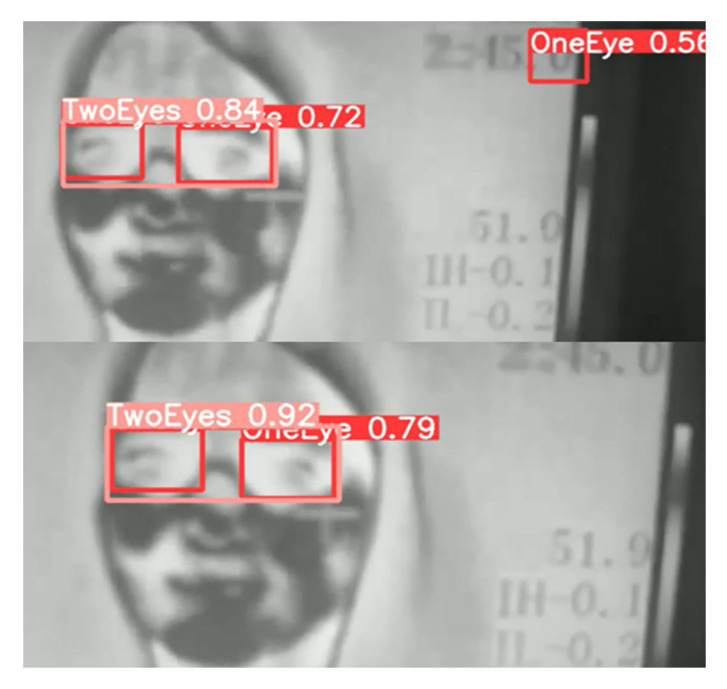
The same video scene with false detection of the one eye class by YOLOv5n, which YOLOv5s does not detect. IoU scores are displayed (Pink boxes are for Two Eyes detection and red boxes are for One Eye detection).

On the other hand, comparing the scenes between the different models’ detections allows us to infer their performance. For example, Figure 14 shows how YOLOv5n model falsely detects number 0 as one eye. However, such false detection is not found in YOLOv5s.

**Figure 15:**
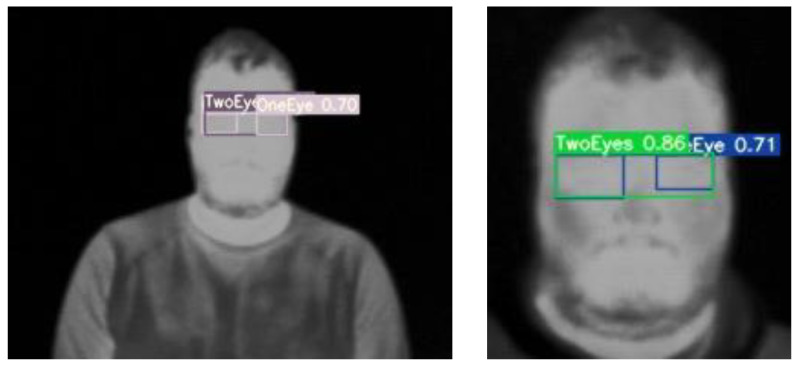
Distance effect on eyes detection by YOLOv7 model trained on the augmented multi-age dataset.

The YOLOv7 model trained on the augmented multi-age dataset is tested for distance effect. A volunteer was asked to stand close to the camera one time, and to move further the other time. The testing performance can be found in Figure 15.

### 3.2. Detection of Inner Canthus Temperature

**Figure 16:**
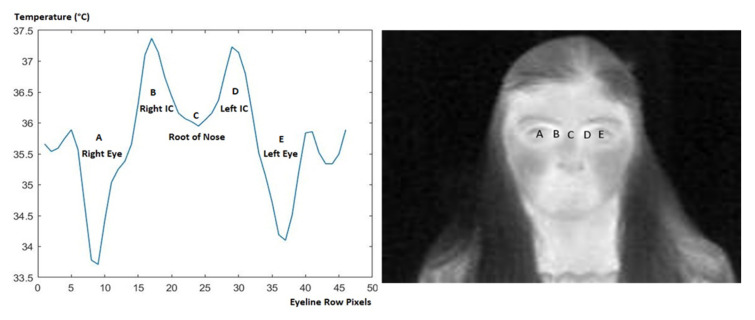
Temperature distribution along eyes line: Temperature of right and left eye, right and left inner canthus (IC) and the root of the nose.

A graphical representation of the temperature distribution along the eyes line passing through both eyes (A and E), the root of the nose (C), and the left, and right inner canthi (IC) (B and D) is presented in Figure 16. In this example, the temperatures of both eye surfaces are 33.7 °C and 34.1 °C, respectively. These temperatures lie in the range of average eye surface temperature (34.51 °C ± 0.82 °C) indicated in the study of Tkáčová et al. [39]. Additionally, we can notice that the temperature significantly rises in the regions of both inner canthi, reaching 37.37 °C and 37.23 °C, respectively. It is important to mention that according to [8], a temperature of 37.5 °C and above is considered fever. The decrease in the temperature at the root of nose is due to the presence of the nasal bone directly underneath the skin and not to blood capillaries [40].

**Figure 17:**
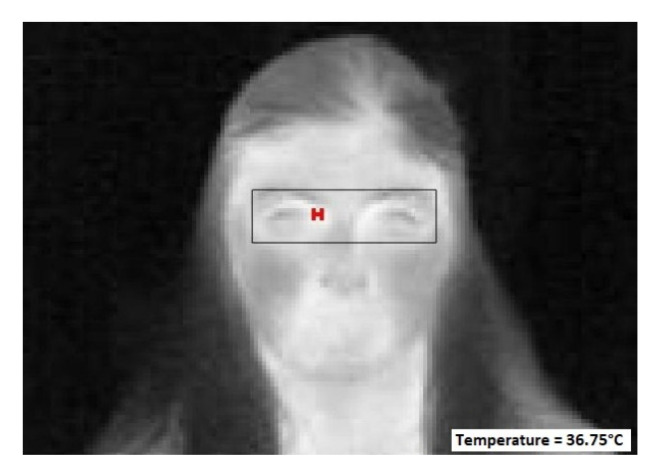
Sample of the temperature data of an image and its detected inner canthus temperature, where the red * (H) belongs to the pixel of the inner canthus detected.

The result of detecting the inner canthus temperature and displaying it on the image along with the bounding box is implemented in MATLAB R2021a and shown in Figure 17. Now, in some cases, the inner canthi of both eyes are detected simultaneously since they both have exactly the same temperature. The process of detecting the inner canthus temperature requires around 0.09 s. The normalized average distance between the automatically detected inner canthus point and the manually located $\left(d\right)$ is calculated using Equation (7), and found to be equal to 0.03.

## 4. Discussion

As shown in Table 1, the increase in the %mAP@0.5 is not significant between the augmented multi-age YOLOv5 models and YOLOv7 (it only increases by 0.1%). However, the major difference among the models is in the FPS, which increased from 58 in YOLOv5n to 115 in YOLOv7, as presented in Table 2. The best-performing model is YOLOv7, with a %mAP@.5 of 99.6% and a speed of 150 FPS. This indicates an accurate and fast eye and inner canthus detection model compared to the ones presented in the literature.

The augmented multi-age model’s experimental results in Table 1 show that the %mAP@.5 score is constant between the different model sizes of YOLOv5. However, when testing a particular image on both YOLOv5n and YOLOv5s, YOLOv5s proved its enhanced performance (Figure 14).

**Figure 18:**
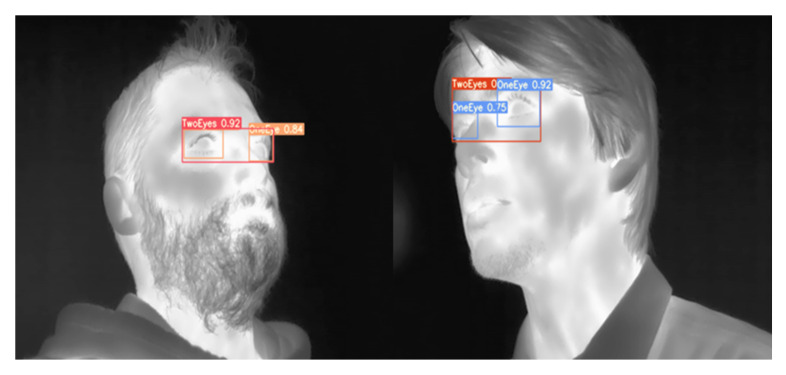
Detection of Eyes in Rotated Faces of the Dataset.

Our method presents a fast and reliable model that accurately detects the eye region. The model is set after training and testing different YOLO versions using different datasets. The multi-age dataset is a combination of our own collected dataset and the online TFW dataset with the application of several augmentation techniques (1827 images). Nevertheless, the elderly dataset is a combination of images from three different datasets: TFW, Tufts, and IRDatabase (656 images). This provides the most diverse and large thermal faces dataset in the literature, with age classification. Our model results in precision and recall equal to 100%. However, the second largest thermal face dataset found in the literature (1000 images) results in precision and recall of 98.8% and 92.6%, respectively, when fed to a cascade classifier after extracting HoG features [12]. Besides the size of our used dataset, it also contains different face poses, enabling the model to identify the eye region in straight-facing images and in rotated-facing images (Figure 18). This is considered a limitation in the system developed by Lazri et al. [18]. In the case of the rotated photos, the detection of the temperature of one inner canthus is sufficient to measure the person’s temperature. This is because, as shown in the graph of Figure 16, there is only a small variation between the temperatures of each inner canthus (0.14 °C).

The augmented multi-age YOLOv7 model successfully detects the eyes in a near face and a far face image, as presented in Figure 17.

In our future work, we aim to accomplish the early detection of infectious diseases by testing for other symptoms such as cough and fatigue. This is in order to raise the alarm when one of these symptoms are present, and undertake the necessary lab tests and corrective actions. Therefore, we have worked on building eye detection models exclusively for seniors, because the appearance of the face landmarks including the eyes changes with age [41]. Elderly images gathered from three different datasets were used to train these models.

In order to test the effect of dataset size on model performance, we trained one model using the original elderly dataset and another one using the augmented elderly dataset. Both trainings started from original YOLOv7 weights. Table 1 shows that the dataset augmentation increases in %mAP@.5 by 0.3%. This is expected, since better deep learning models are built using augmented datasets, as mentioned in [26].

Another approach in establishing the elderly eye detection model is the application of transfer learning to the augmented multi-age YOLOv7 model. This is considered beneficial since it uses the knowledge of somehow similarly pre-trained models to train and fine-tune the new model, which leads to better performance [33]. Table 1 shows how the model trained on the original elderly dataset without augmentation, starting from the weights of YOLOv7 augmented multi-age dataset, performed better (99.5%) than the mode trained starting from the original YOLOv7 weights (99.3%). However, upon using the augmented elderly dataset, there was no difference observed in the %mAP@.5 scores between both models. Furthermore, the results of blindfold testing are promising (99.6%); this proved the generality of the model and its ability to perform well in real case scenarios wherein new individuals are going to be presented to the model.

**Figure 19:**
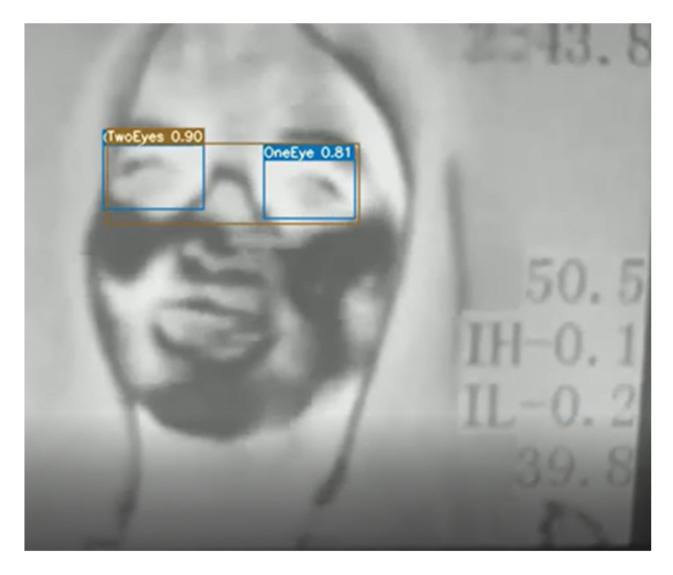
Detection of eyes in video scene by YOLOv7 model with confidence scores.

Our model detects the inner canthus with an acceptable normalized distance of 0.03 with the manually detected method. Despite this shift, we are still in the region of inner canthus and managed to detect the highest temperature in that region, as presented in Section 3 (Figure 17). Additionally, our work could be implemented in a real-time temperature monitoring system, since we were able to detect the eyes in a video scene with a high speed, reaching 150 FPS in the trained YOLOv7 model, as shown in Figure 19. This is compared to a maximum of 146 FPS in [18] when testing a pre-trained single shot multi-box detector (SSD). Regarding accuracy, a comparison between our model and that of [18] is inapplicable, since there, the SSD model is pre-trained on visible images and IoU was as an accuracy metric, whereas our model is trained solely on thermal images, with %mAP@0.5 taken as an accuracy measure.

In general, the limitation of thermal image-based temperature detection systems is their inability to detect the inner canthus temperature in the presence of eye glasses due to their reflective property [15]. The solution would be training a model that detects first the face and the presence of glasses, then searches for the highest temperature in the face outside the eye region. However, this method shall still give an approximation of the body’s temperature rather than the accurate one.

## 5. Conclusions and Perspectives

This paper presents a fast and reliable model that accurately detects the eye region after training and testing different YOLO versions using multi-age and elderly thermal image datasets. Recruiting a thermal camera-based system protects the user’s privacy and is a cheaper solution than visible cameras. This proposed system also allows the comparison of size, speed and accuracy between different recently released object detection YOLO versions: YOLOv5, v6 and v7. In addition, the presence of different head poses in the training dataset and the implementation of data augmentation methods allow the model to detect the eyes and temperature of a person in different orientations. With the aim of frailty and infectious diseases detection, our approach is the first to gather images of the elderly exclusively to build a temperature detection model. The proposed system could be considered a solution for speed, accuracy, and image condition limitations in temperature detection methods, especially during an epidemic. It also can be adapted by hospitals, retirement homes, and homes.

Looking ahead, further studies will be done in order to ensure the accurate measurement of temperature by considering emissivity, distance effect, camera angle and face angle effects, as well as the impact of ambient temperature [20]. Additionally, we aim to implement this work in real-time, due to the high speed of YOLOv7, in order to allow the installation of the system in infirmaries and elderly care homes. This will be done after implementing an alarm system which will be raised when fever is detected. Moreover, although the databases used are considered various and have inter and intra differences, it is important to include a more significant number of individuals to produce more generalized model. Additionally, it is possible to define models for different narrowed age groups that could help integrate our solution in workplaces, schools and nurseries.

## Figures and Tables

**Table 1:** Elderly dataset sources’ distribution.

Dataset Name	Number of Individuals	Number of Images
TFW	7	504
Tufts	11	97
IRDatabase	2	55
Total	20	656

**Table 2:** Precision (P), recall (R) and mAP@.5 Results.

Model Weights	Training Dataset	P	R	F1-Score	%mAP@.5
YOLOv5n	Augmented multi-age dataset	1	1	1	99.5
YOLOv5s		0.99	1	0.99	
YOLOv5m		1	1	1	
YOLOv5l		1	1	1	
YOLOv6n		1	1	1	99.48
YOLOv6s		1	1	1	
YOLOv7		1	0.99	0.99	99.6 ^1^
YOLOv7	Original elderly dataset	1	0.98	0.98	99.3
	Augmented elderly dataset	1	1	1	99.6
YOLOv7Augmented Multi-age Dataset Model Weights	Original elderly dataset	1	1		99.5
	Augmented elderly dataset	1	1		99.6 ^1^
	Blindfold elderly testing dataset				99.6 ^1^

^1^ Highest %mAP@.5.

**Table 3:** The sizes, inference and FPS of different models.

Model	Model Size (MB)	Inference Time	Frames Processed Per Second (FPS)
YOLOv5n	3.6	17.3	58
YOLOv5s	13.6	13.9	72
YOLOv5m	40.1	10	100
YOLOv5l	88.4	8.7	115
YOLOv7	11.68 ^1^	6.7	150 ^2^

^1^ Average size; ^2^ highest FPS.

## Data Availability

The TFW, Tufts, and IRDatabase used and analyzed in this study can be found at http://tdface.ece.tufts.edu/ (accessed on 30 September 2022), https://github.com/IS2AI/TFW (accessed on 5 July 2022), https://github.com/marcinkopaczka/thermalfaceproject (accessed on 25 April 2022).
